# Mouse Models of Diabetes, Obesity and Related Kidney Disease

**DOI:** 10.1371/journal.pone.0162131

**Published:** 2016-08-31

**Authors:** Sarah J. Glastras, Hui Chen, Rachel Teh, Rachel T. McGrath, Jason Chen, Carol A. Pollock, Muh Geot Wong, Sonia Saad

**Affiliations:** 1 Department of Medicine, Kolling Institute, University of Sydney, Sydney, Australia; 2 Department of Diabetes, Endocrinology and Metabolism, Royal North Shore Hospital, St Leonards, NSW 2065, Australia; 3 School of Life Sciences, Faculty of Science, University of Technology Sydney, Sydney, Australia; 4 Department of Anatomical Pathology, Royal North Shore Hospital, St Leonards, NSW, Australia; University of Louisville, UNITED STATES

## Abstract

Multiple rodent models have been used to study diabetic kidney disease (DKD). The purpose of the present study was to compare models of diabetes and obesity-induced metabolic syndrome and determine differences in renal outcomes. C57BL/6 male mice were fed either normal chow or high fat diet (HFD). At postnatal week 8, chow-fed mice were randomly assigned to low-dose streptozotocin (STZ, 55 mg/kg/day, five consecutive days) or vehicle control, whereas HFD-fed mice were given either one high-dose of STZ (100 mg/kg) or vehicle control. Intraperitoneal glucose tolerance tests were performed at Week 14, 20 and 30. Urinary albumin to creatinine ratio (ACR) and serum creatinine were measured, and renal structure was assessed using Periodic Acid Schiff (PAS) staining at Week 32. Results showed that chow-fed mice exposed to five doses of STZ resembled type 1 diabetes mellitus with a lean phenotype, hyperglycaemia, microalbuminuria and increased serum creatinine levels. Their kidneys demonstrated moderate tubular injury with evidence of tubular dilatation and glycogenated nuclear inclusion bodies. HFD-fed mice resembled metabolic syndrome as they were obese with dyslipidaemia, insulin resistance, and significantly impaired glucose tolerance. One dose STZ, in addition to HFD, did not worsen metabolic features (including fasting glucose, non esterified fatty acid, and triglyceride levels). There were significant increases in urinary ACR and serum creatinine levels, and renal structural changes were predominantly related to interstitial vacuolation and tubular dilatation in HFD-fed mice.

## Introduction

Diabetes is the leading cause of chronic kidney disease (CKD) worldwide not only in Western countries, but also in low- or middle-income countries [1]. Type 2 diabetes mellitus (T2D), together with its related kidney disorders, is increasing in pandemic proportions largely driven by the exponential rise in obesity [2,3]. An important and growing complication of T2D is diabetic kidney disease (DKD) [4,5]. Moreover, obesity itself has been shown to have detrimental effects on renal outcomes independent of diabetes [6–8]. Advanced CKD is associated with a huge personal burden given that kidney transplantation and dialysis are the only options for late stages of CKD, and these renal replacement therapies are costly and have significant ramifications for healthcare expenditure [9]. Hence, a greater understanding of CKD and how it relates to diabetes and obesity is imperative, in order to devise better diagnostic and treatment strategies for the prevention and treatment of CKD.

In humans, renal biopsy is invasive and costly and is not routinely performed in patients with known diabetes, obesity and renal functional changes. Although DKD is typically associated with the histological features of diabetic nephropathy including diffuse mesangial thickening, glomerular basement membrane thickening and tubulointerstitial fibrosis, a significant portion of individuals with diabetes and impaired renal function do not have classic histological signs of diabetic nephropathy [10]. Classic renal features associated with severe obesity in humans include glomerulopathy and focal segmental glomerulosclerosis (FSGS) [11]. However, FSGS associated with extreme obesity alone is quite uncommon whereas renal damage in the context of obesity together with other features of the metabolic syndrome is frequently seen [2,12]. In order to study obesity-induced renal disease mimicking human pathophysiology, it is important to establish animal models of obesity together with glucose dysregulation, hyperlipidaemia, and hypertriglyceridaemia.

Compared to human studies, animal models have the advantage of controlled experimental designs to systematically assess the value of therapeutic interventions. Consumption of a high-fat diet (HFD) in rodents, sheep and primates typically yields obesity [13]. The C57BL/6 mouse model is advantageous given its short gestational period and long lifespan, ease of availability and the animal’s tendency to over-consume HFD, thus mimicking human behaviour resulting in an obese phenotype. Furthermore, the wild type C57BL/6 mouse is particularly susceptible to weight gain when fed HFD compared to other genetically manipulated mouse models [14]. Over time, C57BL/6 mice typically exhibit features commonly associated with the complex metabolic syndrome in humans, which include obesity, insulin resistance, glucose intolerance, hyperlipidaemia, hypertriglyceridaemia and hypertension [15,16]. They are susceptible to glucose intolerance, non-alcoholic fatty liver disease and endothelial damage commonly associated with cardiovascular disease [15–17]. With respect to renal outcomes, mice on a HFD showed albuminuria, glomerulomegaly, mesangial expansion, increased type IV collagen, renal lipid accumulation, increased macrophage infiltration and elevated markers of oxidative stress [16].

The type of diabetes that is of interest should influence the animal model utilised to study DKD. In comparison to T2D with its associated metabolic features, type 1 diabetes mellitus (T1D) is an autoimmune form of diabetes characterised by progressive destruction of the pancreatic beta cell and insulin deficiency [18]. Relative beta cell depletion and loss of beta cell function are also thought to be important factors in the development of T2D, although in the case of T2D, the process is not thought to be autoimmune in nature [19]. One method of inducing diabetes using a wild-type animal is by administering streptozotocin (STZ), a pharmacological agent that induces beta cell destruction by intracellular alkylation of DNA and subsequent beta cell necrosis [20–22]. There is variability in the timing, dosage and frequency of STZ injections and different protocols can be employed to mimic T1D or T2D in a variety of species/strains [20,23–25]. The mouse model utilising five doses of STZ at low dose (50–60 mg/kg/day) has been extensively used to mimic T1D due to the progression destruction of beta cell mass [20]. In comparison, HFD together with one high dose of STZ has been suggested as a useful model of T2D, encompassing both the features of obesity and insulin resistance due to HFD and moderate beta cell reduction [26]

In the present study, we aimed to characterise the metabolic and renal outcomes in a mouse model of insulin deficiency mimicking T1D and diet-induced obesity associated with metabolic dysregulation representing T2D. Specifically, we compared the metabolic features and renal outcomes in a C57BL/6 mouse model using five low doses of STZ (55 mg/kg/day), HFD alone or HFD together with one high dose of STZ (100 mg/kg).

## Materials and Methods

### Animal experiments

**Fig 1:**
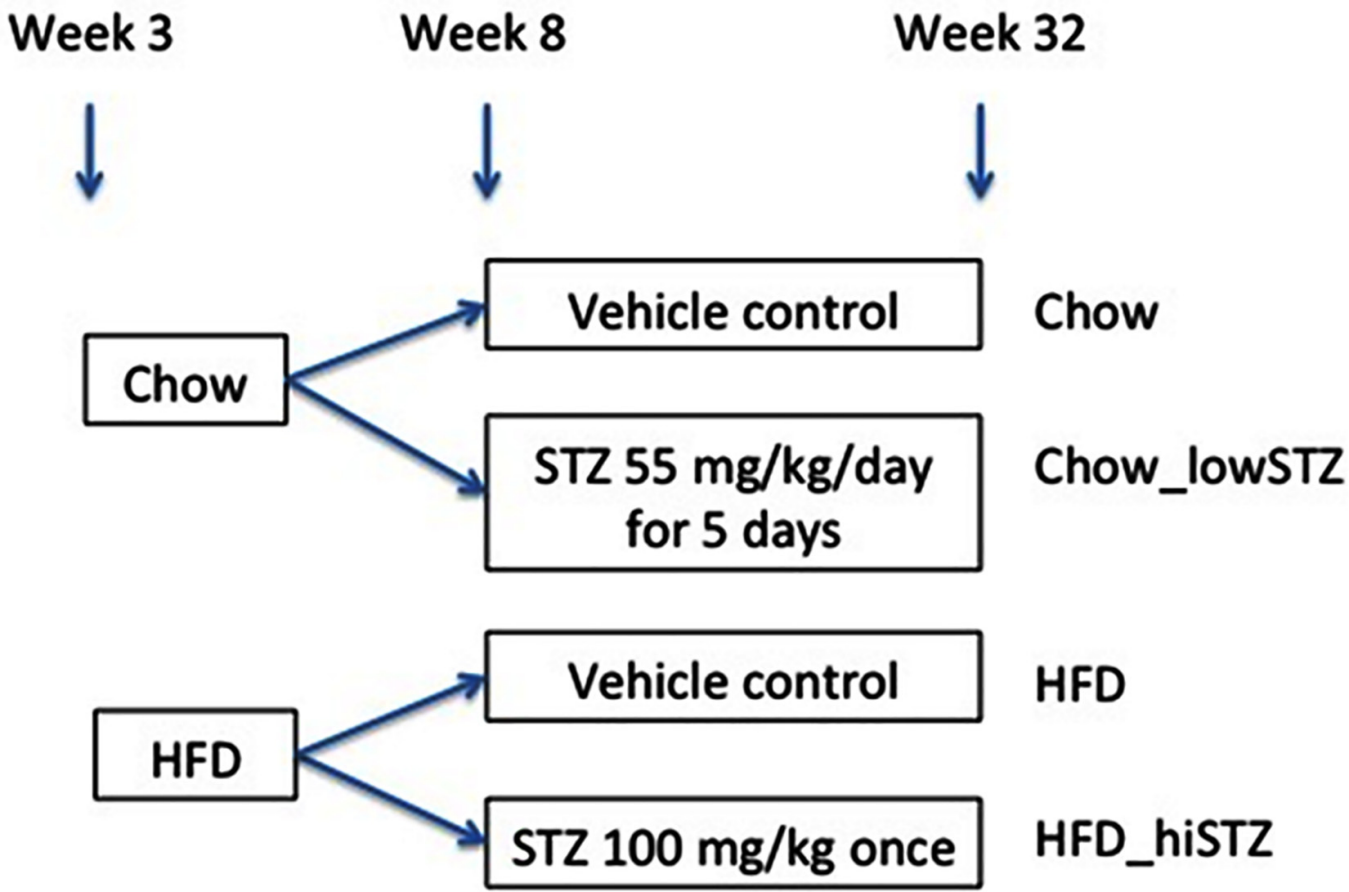
Schematic representation of the animal model utilised in this study. Chow represents control group fed normal chow diet; Chow_lowSTZ depicts the group exposed to normal chow diet and low-dose streptozotocin (STZ) for five consecutive days; HFD depicts the group fed HFD; HFD_hiSTZ depicts the group fed HFD in conjunction with high-dose STZ (one dose only).

The animal models utilised in the present study included a control group fed normal chow diet (Chow group), a group fed chow diet and given five low doses of STZ (Chow_lowSTZ group), a group fed HFD (HFD group) and a group fed HFD together with a single high dose of STZ (HFD_hiSTZ group). A schematic representation of the animal model used in this study is presented in Fig 1. All animals were housed in the Kearns Animal Facility of Kolling Institute, Royal North Shore Hospital with a stable environment maintained at 22±1°C with a 12/12-h light–dark cycle. All procedures were approved by the Animal Care and Ethics Committee (AEC) of Royal North Shore Hospital (AEC 1309-007A) and complied with the Australian Code of Practice for the Care and Use of Animals for Scientific Purposes.

Male pups were weaned from C57Bl/6J dams (sourced from Kearns Facility, Kolling Institute, Royal North Shore Hospital, St Leonards, NSW, Australia) at postnatal day 20 (normal weaning age) and fed standard rodent chow (11kJ/g, 14% fat, Gordon’s Specialty Stockfeeds, NSW, Australia) or pellet HFD (20kJ/g, 43% fat; SF03-020, Speciality Feeds, WA, Australia). At postnatal week 8, mice fed chow diet were assigned to either low-dose streptozotocin (STZ, 55 mg/kg, ip for five consecutive days) or vehicle control (citrate buffer) whereas mice fed HFD were assigned to either high-dose STZ (100 mg/kg, i.p., once) or vehicle control. To deliver STZ, mice were fasted for 5 h prior to injection. STZ (in 0.1 M citrate buffer, pH 4.5, Sigma, MO, USA) was freshly prepared.

The frequency of animal monitoring was once per fortnight. Animal health, body condition and wellbeing were assessed each time. No adverse events occurred during the experiments described. Mice were weighed fortnightly and their blood glucose levels were measured using an Accu-Chek glucometer (Roche Diagnostics) following 6 h fasting period. Only animals with fasting blood glucose >16 mmol/L were considered to be diabetic. Diabetic mice received insulin (2U glargine, Germany) to prevent ketosis if their blood glucose level was above 25 mmol/L and subsequently blood glucose readings were performed twice weekly. Euthanasia was carried out by deep anaesthesia with isoflurane (4%) followed by cardiac puncture. Tissue harvesting took place at Week 32 under fasting conditions. Organ perfusion was performed with PBS after cardiac puncture for blood collection. The kidneys, liver, and fat were collected and weighed then the kidney was fixed in 10% buffered formalin for histological examination.

Mice were placed in metabolic cages and 24-h urine collection was performed one week prior to sacrifice. Urine albumin levels were determined using the Murine Microalbuminuria ELISA kit (Exocell, Inc., Philadelphia, PA, USA) and urine creatinine levels were determined using the Microcreatinuria ELISA kit (Exocell, Inc., Philadelphia, PA, USA).

Intraperitoneal glucose tolerance tests (IPGTTs) were performed at Week 14, Week 20 and Week 30. Mice were fasted for at least 6 h prior to the test. At baseline, the blood glucose level was measured. Glucose was administered at Time 0 (2 g/kg, Phebra, Australia) and then blood glucose levels recorded at Time 15 min, 30 min, 60 min and 90 min. Animals were fully conscious throughout the IPGTT.

### Bioassays

Glycosylated haemoglobin (HbA1c) was measured using a DCA Vantage Analyzer (Siemens Medical Solutions Diagnostics, Tarrytown, NY) [27]. Serum creatinine and lipid profile including total serum cholesterol, triglycerides and LDL were measured using the Architect C16000 Clinical Chemistry Analyzer (Abbott Laboratories, Abbott Park, Il, USA) available through the affiliated hospital pathology service. Plasma non-esterified fatty acids (NEFA) were measured using a NEFA kit (WAKO, Osaka, Japan) [13]. Serum insulin was measured using a mouse-specific ELISA method (Merck, Darmstadt, Germany). The density was detected on a Bio-Rad 680 XR (Hercules, CA, USA) [13, 18]. HOMA-IR was quantified as blood glucose level multiplied by serum insulin level divided by 22.5.

### Analysis of renal structural changes

Formalin-fixed hemisected kidneys were embedded in paraffin and stained with Periodic Acid Schiff (PAS). Two independent, blinded observers including an anatomical pathologist and a nephrologist performed histological analyses using a light microscope (Olympus photomicroscope linked to a DFC 480 digital camera).

One kidney hemisection was examined at 100x magnification for foci of tubulointerstitial fibrosis and graded using a scale of 0 to 4 (0—normal; 1—involvement of < 10% of the cortex; 2—involvement of 10–25% of the cortex; 3—involvement of 25–75% of the cortex; and 4—extensive damage involving > 75% of the cortex). Tubular injury was determined using ten random non-overlapping high power fields (HPFs) of renal cortex at 400x magnification and assessment of four categories of damage was made including: (A) tubular vacuolation, (B) tubular dilatation, C) glycogenated nuclei, and (D) tubular cast. For tubular dilatation: 0, absence, 1, <5 dilated cortical tubules were observed per high power field (HPF), 2, 5–10 dilated cortical tubules were observer per HPF, 3, >10 dilated tubules were observed per HPF. For tubular vacuolation: 0, absence, 1, <25% of the cortical tubules have vacuoles 2, 25–50% cortical have vacuoles, 3, >50% cortical tubules have vacuoles. For glycogenated nuclei in tubular epithelium, 0, absence, 1, only 1 glycogenated nuclear observed per HPF; 2- 2to 3 nuclei observed per HPF, 3, > 3 glycogenated nuclei observed per HPF. For cast appearance, 0, absence, and 1, present.

For glomerulosclerosis, the first 20 randomly selected glomeruli at the kidney cortex were examined and graded as previously described [28,29]. The sections were scored as 0—normal, 1—< 25% involvement, 2 < 50% involvement, 3—< 75%, and 4—> 75% sclerosis and then the average of 20 individual scores was calculated to generate the glomerulosclerosis score

### Statistical methods

All results are expressed as mean ± SEM. Data were analysed using analysis of variance (ANOVA), followed by post hoc Bonferroni tests when the difference between groups was being considered. The trapezoidal rule was used to determine the area under the curve (AUC) during IPGTT results. For differences in plasma glucose levels during the IPGTT, an ANOVA with repeated measures was performed and significance determined using Tukey’s post hoc test. All analyses were carried out using GraphPad Prism 6.0 (GraphPad Software, San Diego, CA, USA) and a *P* value of < 0.05 was considered statistically significant.

## Results

### Anthropometric parameters

Body weight was expected to be reduced in a model with multiple STZ injections and higher in HFD-induced obesity. The group exposed to five doses of STZ had a significantly lower body weight compared to the control Chow group at Week 32 (P < 0.01, Chow vs. Chow_lowSTZ). Moreover, they showed significantly less weight gain between the induction of diabetes at Week 8 and Week 32 (P < 0.05, Chow_lowSTZ vs. Chow). In addition, three of the 14 animals in the Chow_lowSTZ group lost weight between Week 8 and Week 32. In comparison, both HFD and HFD_hiSTZ groups were significantly heavier compared to the Chow group (P < 0.0001, Chow vs. HFD, and Chow vs. HFD_hiSTZ, Table 1), over the course of 24 weeks concordant with the human obese T2D phenotype. The HFD group was significantly heavier than the HFD_hiSTZ group (P < 0.0001, Table 1).

**Table 1:** Anthropometric measures at 32 weeks of age.

	Chow	Chow_lowSTZ	HFD	HFD_hiSTZ
BW (g)	27.26 ± 0.27	24.02 ± 0.72*	42.06 ± 1.21**	33.02 ± 1.56**^###^
Weight gain (g) from Week 8	5.52 ± 0.40	2.85 ± 0.76*	13.67 ± 1.27**	8.50 ± 1.31^###^
Kidney (g)	0.22 ± 0.01	0.19 ± 0.01	0.29 ± 0.01**	0.24 ± 0.01^#^
Kidney (% BW)	0.80 ± 0.02	0.79 ± 0.03	0.74 ± 0.02	0.77 ± 0.05
Liver (% BW)	5.40 ± 0.27	5.53 ± 0.14	8.01 ± 0.48**	6.03 ± 0.36^##^
Retroperitoneal fat (% BW)	0.52 ± 0.06	0.64 ± 0.16	2.35 ± 0.28**	1.37 ± 0.22**^##^
Eipdidymal fat (% BW)	1.68 ± 0.10	1.53 ± 0.16	5.34 ± 0.34**	4.24 ± 0.48**^#^

BW: body weight. Compared with control

*P<0.01

**P<0.0001; HFD_hiSTZ compared with HFD

# P < 0.05

## P < 0.001

### P < 0.0001. Results are expressed as mean ± SEM, n = 9–12. Control: Chow; Chow diet and five low-dose STZ: Chow_lowSTZ; High fat diet: HFD; and HFD and one high-dose STZ: HFD_hiSTZ.

To determine the impact of diet and/or STZ treatment on kidney size, the left and right kidney masses were averaged for each animal. Kidney size as percentage of body weight was similar among the groups (Table 1). The liver was significantly heavier in the HFD group compared to the Chow group (P < 0.0001 vs. Chow) and HFD_hiSTZ group (P < 0.001 vs. Chow). As expected in a model of obesity, animals fed HFD had significantly more percentage of fat mass at Week 32 as measured by both retroperitoneal and epididymal fat deposits (P < 0.0001, HFD vs. Chow for both fat measures). There was significantly less fat deposition in the retroperitoneal and epididymal regions in the HFD_hiSTZ versus HFD group, as a consequence of the one dose of STZ at Week 8 presumably due to the adverse effects induced by STZ (P < 0.01, P < 0.05 respectively, Table 1).

### Glucose tolerance test results

To characterise the impact of HFD and exposure to one dose of STZ, glucose tolerance was measured by performing an IPGTT in Chow, HFD and HFD_hiSTZ groups at postnatal Week 14, 20 and 30 (corresponding to 6 weeks, 12 weeks and 22 weeks post STZ injection).

**Fig 2:**
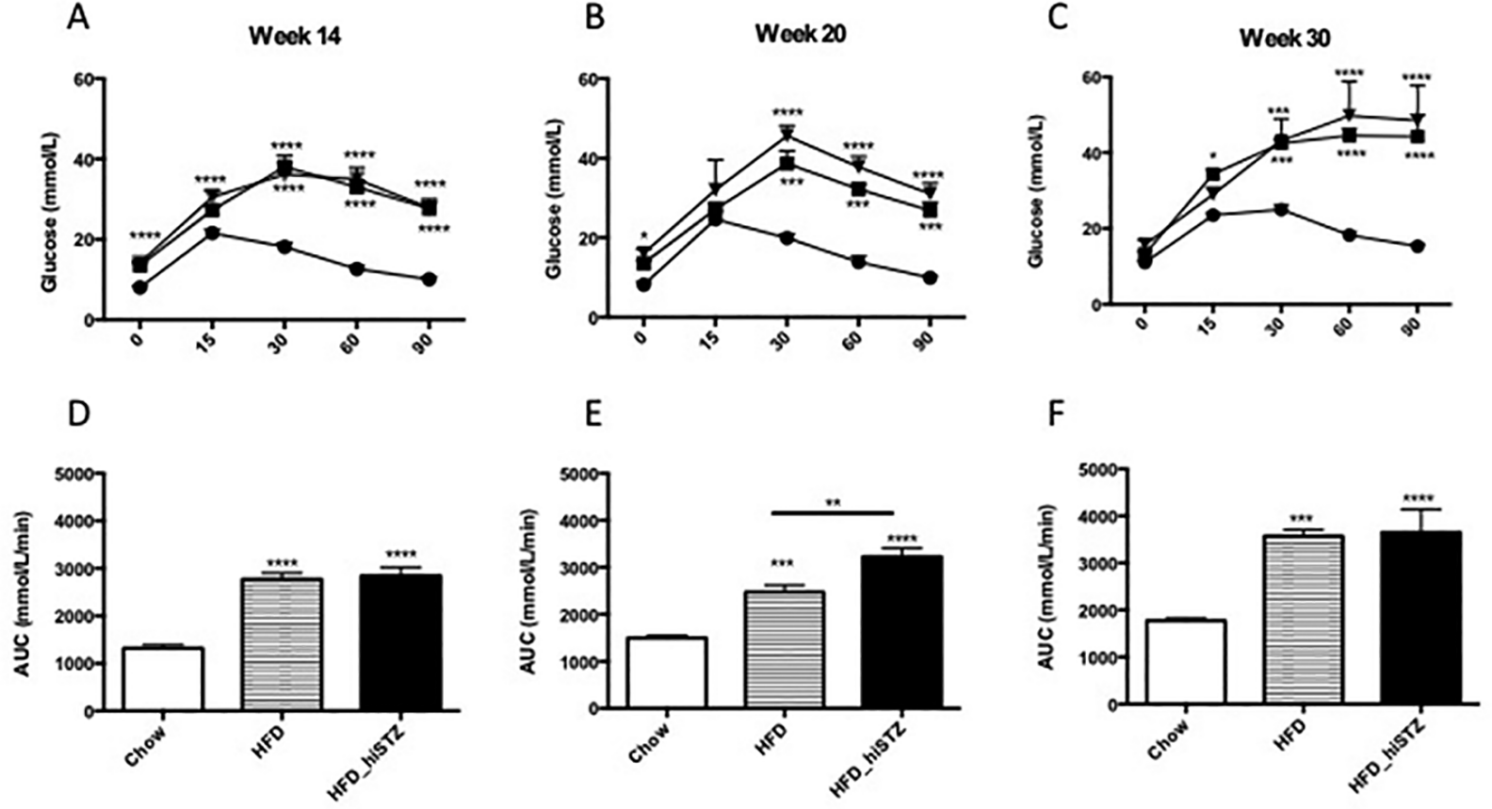
Intraperitoneal glucose tolerance tests performed at postnatal Week 14, 20 and 30 in C57Bl/6J mice. Blood glucose levels were measured at 0,15,30,60 and 90 minutes as seen in A-C. Symbols depicted in A-C: Ctrl, designated with a circle; HFD, designated with a square; HFD-hiSTZ, designated with an inverted triangle. Area under the curve was calculated using the trapezoid rule and is shown in D-F. Results are expressed as mean ± SEM, n = 7–14. *P< 0.05, **P<0.01, ***P<0.001, ****P < 0.0001 compared to control, # compared to HFD. Control: Chow; High fat diet: HFD; and HFD and one high-dose STZ: HFD_hiSTZ.

At Week 14 (6 weeks post-STZ injection), fasting glucose at Time 0 was greater in the HFD and HFD_hiSTZ groups than the Chow group (P < 0.0001, Chow vs. HFD_hiSTZ; Chow vs. HFD, P < 0.0001, Fig 2A). From 15 to 90 minutes, the glucose levels in the HFD_hiSTZ, and HFD groups were significantly higher than the Chow fed mice (P < 0.0001 for both, Fig 2A), with no significant difference between HFD and HFD_hiSTZ at these time points.

At Week 20 (12 weeks post-STZ injection), higher fasting glucose levels were observed at Time 0 in the HFD_hiSTZ group compared to the Chow group (P < 0.05, Fig 2B). There were no differences between the 3 groups at 15 minutes. At Time 30 and beyond, both HFD and HFD_hiSTZ had higher glucose readings in comparison to the Chow fed mice (P < 0001, Chow vs. HFD; P < 0.0001, Chow vs. HFD_hiSTZ, at Time 30, 60 and 90). When the HFD and HFD_hiSTZ groups were compared, there were no significant differences at any time point.

At Week 30 (20 weeks post-STZ injection), both the HFD and HFD_hiSTZ groups still demonstrated significant glucose intolerance compared to the Chow group (Fig 2C). Despite no statistical difference in fasting glucose levels, both groups had higher glucose readings at Time 30, 60 and 90 (P < 0.001, Chow vs. HFD, Chow vs. HFD_hiSTZ, at Time 30; P < 0.0001 at Time 60 and 90). There was no difference in blood glucose level between HFD and HFD_hiSTZ groups at any time point.

The AUC value was elevated at Week 14, Week 20 and Week 30 in both HFD and HFD_hiSTZ groups compared to the Chow group (P<0.0001, Fig 2D–2F). The AUC value was significantly higher in the HFD_hiSTZ versus HFD group at Week 20 but not at Week 14 or Week 30 (p < 0.01, HFD vs. HFD_hiSTZ at Week 20).

Interestingly, at Week 30 in both the HFD and HFD_hiSTZ the shape of the IPGTT curve was dissimilar to the shape of the curve at Week 14 and Week 20 due to persistence of hyperglycaemia and failure to recover normoglycaemia by 90 minutes. It suggests impaired second phase insulin response.

### Serum metabolic measures

Glycosylated haemoglobin (HbA1c) was significantly elevated in the Chow_lowSTZ group alone suggesting that this group had the most significant impairment in glucose control consistent with a model of T1D (P < 0.0001, Chow_lowSTZ vs. Chow; Table 2). Nonetheless, HbA1c was lower in the C57BL/6 diabetic mouse compared to both humans and other mouse strains, consistent with previously described data [27].

**Table 2:** Metabolic measures at 32 weeks of age.

	Chow	Chow_lowSTZ	HFD	HFD_hiSTZ
Fasting Glucose (mmol/L)	14.11 ± 0.47	22.23 ± 1.09***	19.92 ± 0.74 ***	22.14 ± 1.18 ***
HbA1c (%)	4.51 ± 0.07	5.85 ± 0.27***	4.82 ± 0.17	4.67 ± 0.09
Serum insulin (mIU/L)	8.01 ± 1.31	3.38 ± 0.70*	16.89 ± 4.77*	5.99 ± 1.61^###^
HOMA-IR	0.18 ± 0.03	0.14 ± 0.03	0.59 ± 0.17*	0.34 ± 0.09^#^
Serum total cholesterol (mmol/L)	2.33 ± 0.08	2.40 ± 0.19	6.40 ± 0.45 ****	4.42 ± 0.09 ****^####^
Serum triglycerides (mmol/L)	0.70 ± 0.12	0.50 ± 0.15	1.08 ± 0.13*	0.84 ± 0.10
Serum LDL (mmol/L)	0.60 ± 0.07	0.88 ± 0.07	3.25 ± 0.38 ****	1.57 ± 0.20 *^####^
Serum NEFA (mmol/L)	0.85 ± 0.04	0.95 ± 0.06	1.10 ± 0.07**	1.00 ± 0.05*

* P < 0.05

** P < 0.01

*** P < 0.001

****P<0.0001 Compared with control

# P < 0.05

### P < 0.001

#### P < 0.0001 HFD_hiSTZ compared with HFD. Results are expressed as mean ± SEM, n = 6–12. Control: Chow; Chow diet and five low-dose STZ: Chow_lowSTZ; High fat diet: HFD; and HFD and one high-dose STZ: HFD_hiSTZ.

Fasting insulin levels in the Chow_lowSTZ group were significantly lower compared to Chow (P < 0.05, Table 2), likely due to STZ-induced destruction of pancreatic β-cells similar to T1D. In the HFD group, there was evidence of fasting hyperinsulinaemia (P < 0.01, HFD vs. Chow), and insulin resistance measured by HOMA-IR was increased (HFD vs. Chow P < 0.01). There was no difference in insulin levels or HOMA-IR between the HFD_hiSTZ and Chow group at Week 32. Interestingly serum insulin and HOMA-IR were two to three fold lower in the HFD_hiSTZ versus HFD groups, presumably as a consequence of one high dose of STZ at Week 8 (P < 0.05 respectively).

Hyperlipidaemia was assessed by measuring fasting total serum cholesterol, triglycerides, low-density lipoproteins (LDL), and NEFA. Total cholesterol was significantly elevated in the HFD and HFD_hiSTZ groups (HFD vs. Chow and HFD_hiSTZ vs. Chow < 0.0001, Table 2). Fasting total cholesterol was higher in the HFD versus HFD-hiSTZ groups (P < 0.0001). Likewise, serum LDL level was markedly higher in the HFD versus Chow group by five folds (P < 0.0001) and also elevated in the HFD_hiSTZ group compared to the Chow group (P < 0.01). However, the LDL concentration in the HFD_hiSTZ group was significantly lower than that in the HFD group (HFD_hiSTZ vs. HFD, P < 0.0001). Furthermore, the levels of NEFA were raised in the HFD and HFD_hiSTZ groups (P<0.01, P< 0.05 respectively). There was no difference in either triacylglycerol or NEFA between HFD and HFD_hiSTZ groups.

As anticipated, in each of the three models of diabetes/obesity, fasting glucose was significantly elevated compared to Chow control at Week 32 (Chow_lowSTZ, HFD, HFD_hiSTZ vs. Chow P < 0.0001, Table 2). Interestingly, there were no significant differences in fasting glucose levels between any of the three models.

### Measures of renal function

**Fig 3:**
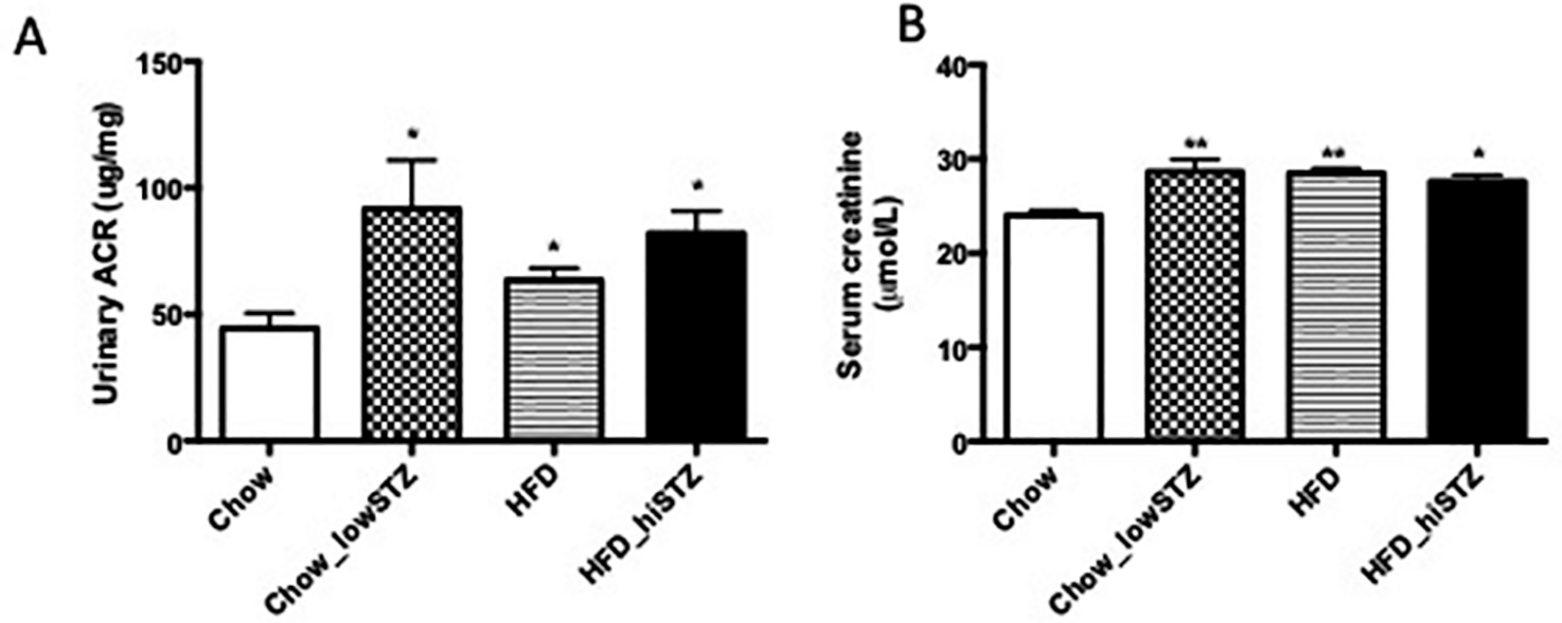
Renal functional changes demonstrated at postnatal Week 31 and 32. (A) Urinary albumin to creatinine ratio (ACR) collected at Week 31 from metabolic cage, (B) Serum creatinine at Week 32. Results are expressed as mean ± SEM, n = 9 for ACR, n = 6–9 for 24 h albumin. *P< 0.05, **P<0.01 compared to Ctrl; # P < 0.05 compared to Chow_lowSTZ. Control: Chow; Chow diet and five low-dose STZ: Chow_lowSTZ; High fat diet: HFD; and HFD and one high-dose STZ: HFD_hiSTZ.

The urinary ACR was measured after collecting urine for 24 h whilst mice were placed in a metabolic cage. Urinary ACR was elevated in the Chow_lowSTZ group compared to the Chow controls (P < 0.05, Fig 3A). Additionally, both HFD and HFD_hiSTZ groups had higher ACR compared to the chow-fed mice (HFD, HFD_hiSTZ vs. Chow, P < 0.05). To further examine renal function, serum creatinine was measured and importantly was elevated in all three groups of diabetes/obesity at Week 32 (Chow_lowSTZ, HFD, HFD_hiSTZ vs. Chow, P < 0.05, Fig 3B).

### Renal structural changes

**Fig 4:**
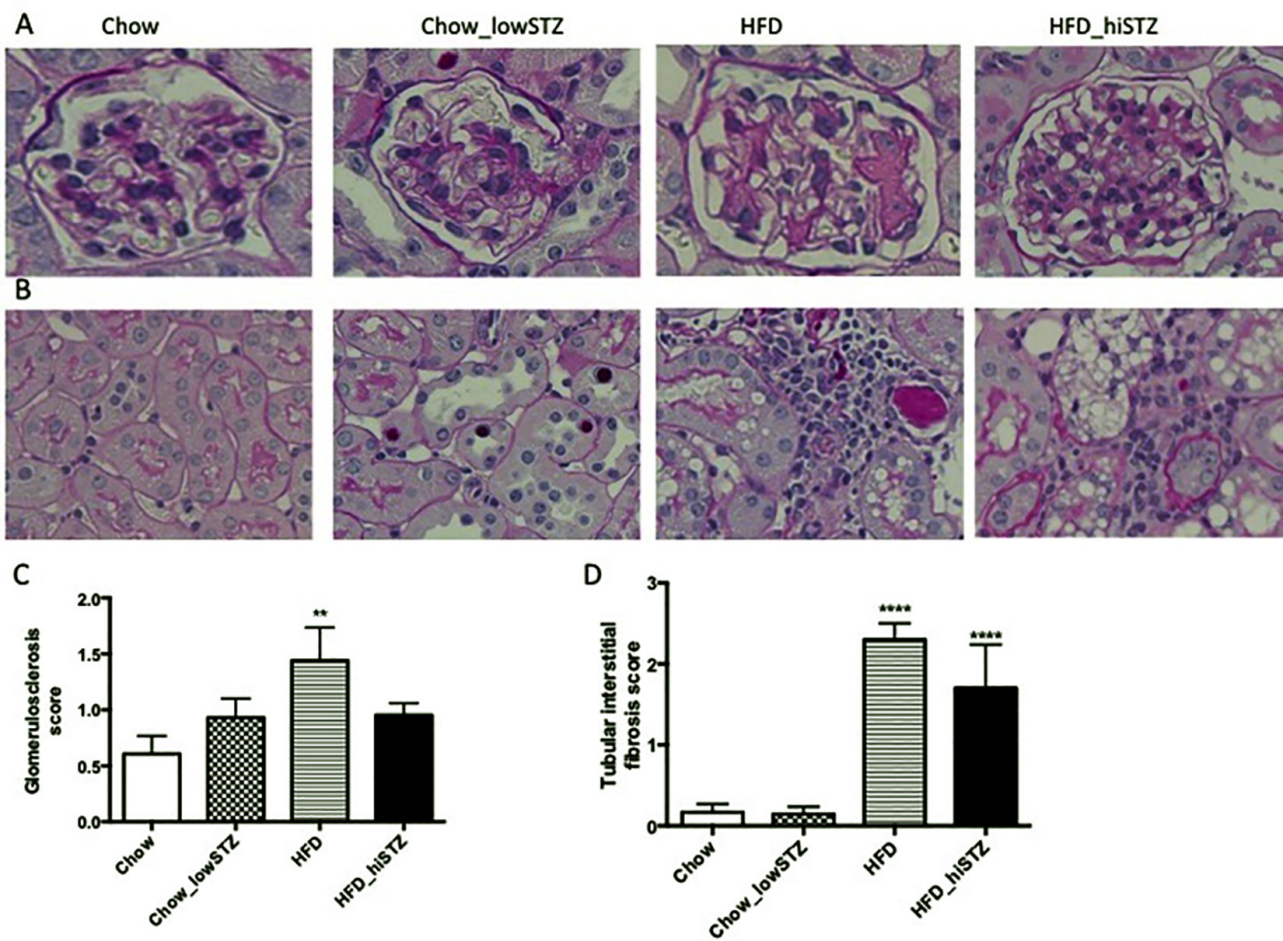
Periodic acid Schiff (PAS) staining at Week 32. (A) Representative images at high magnification of glomerular changes, (B) Representative images at high magnification of tubular damage, (C) Tubular interstitial fibrosis score, and (D) Glomerulosclerosis score. Results are expressed as mean ± SEM, n = 6. *P< 0.05, **P<0.01 compared to Chow_lowSTZ. Control: Chow; Chow diet and five low-dose STZ: Chow_lowSTZ; High fat diet: HFD; and HFD and one high-dose STZ: HFD_hiSTZ.

Glomerulosclerosis was demonstrated by PAS staining which contributed to a higher glomerulosclerosis index score in the HFD group (HFD vs. Chow, p < 0.05, Fig 4A and 4C). There were no differences in the glomerulosclerosis score between Chow_lowSTZ or HFD_hiSTZ versus Chow group. For the purpose of this study, tubulointerstitial fibrosis is strictly referred to as a combination of widening of the interstitium, accumulation of inflammatory cells, tubular atrophy, and wrinkling and/or thickened of the tubular basement membrane. Perivascular areas were excluded during scoring. Tubulointerstitial fibrosis was evident in the HFD-fed groups (HFD, HFD_hiSTZ vs. Chow, P < 0.0001, Fig 4B and 4D). Interestingly, there was no difference in the tubulointerstitial fibrosis score seen between Chow_lowSTZ and Chow.

**Fig 5:**
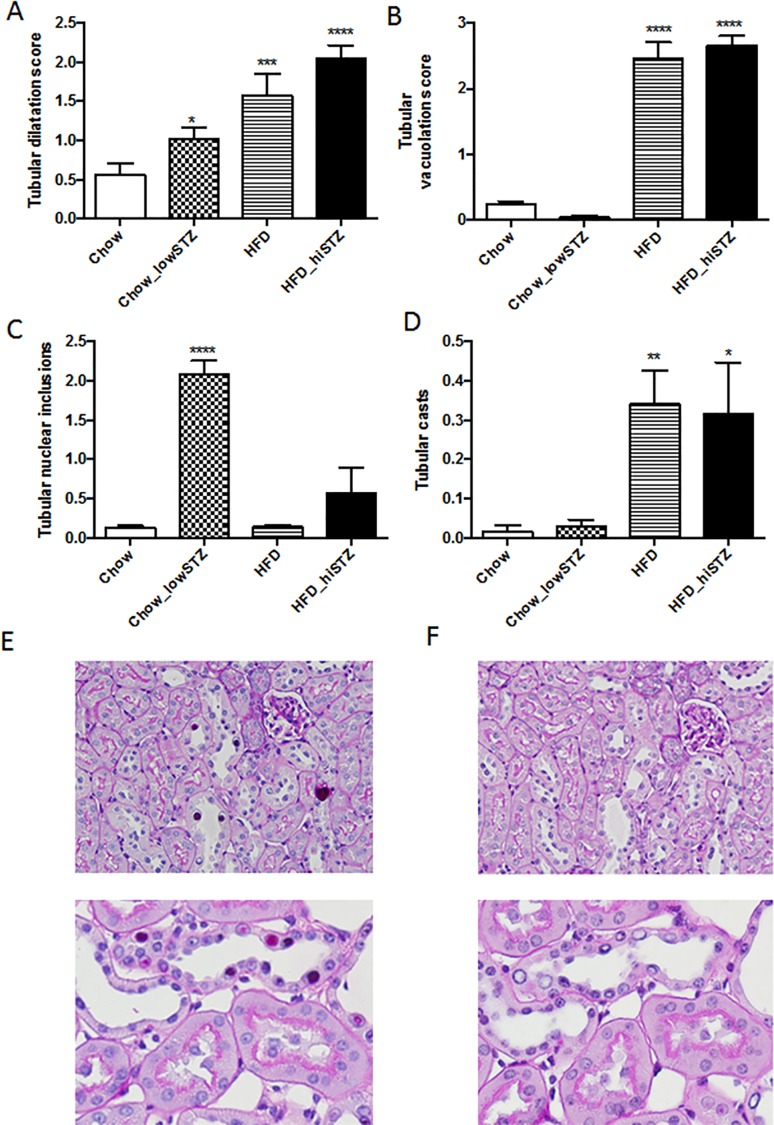
Periodic acid Schiff (PAS) staining was used to demonstrate tubular injury at Week 32. Tubular injury was scored according to: (A) Tubular dilation, (B) Tubular vacuolation, (C) Glycogenated nuclei, and (D) Tubular casts. (E) Representative image of nuclear inclusion bodies (due to glycogenation) with PAS, (F) Representative image of the absence of glycogenated nuclei with PAS and diastase (an enzyme used to degrades glycogen). Results are expressed as mean ± SEM, n = 6. *P< 0.05, **P<0.01 compared to Chow_lowSTZ. Control: Chow; Chow diet and five low-dose STZ: Chow_lowSTZ; High fat diet: HFD; and HFD and one high-dose STZ: HFD_hiSTZ.

The Chow_lowSTZ group demonstrated features of tubular injury including tubular dilatation (Chow_lowSTZ vs. Chow, P < 0.05, Fig 5A) and glycogen intranuclear inclusions within the nuclei of the proximal tubules (Chow_lowSTZ vs. Chow, P < 0.0001, Fig 5C). To confirm that the intranuclear inclusions were glycogenated, PAS staining was performed with or without the addition of diastase, an enzyme that breaks down glycogen. With the addition of diastase, the intranuclear inclusions were no longer observed confirming the content of the intranuclear inclusions as glycogen (Fig 5E and 5F). There was minimal evidence of glycogen intranuclear inclusions in the proximal tubules of the HFD or HFD_hiSTZ groups. In contrast, the most striking features in the kidneys of the HFD and HFD_hiSTZ groups were tubular vacuolation and marked tubular dilatation (vacuolation score for HFD or HFD_hiSTZ vs. Chow, P < 0.0001 and dilatation score for HFD or HFD_hiSTZ vs. Chow, P < 0.001 and P < 0.0001 respectively, Fig 5A and 5B). There was no difference between the tubular vacuolation or dilatation scores between the HFD and HFD_hiSTZ groups. Tubular casts were also present in the HFD and HFD_hiSTZ groups whereas they were barely noticed in either the Chow or Chow_lowSTZ groups (HFD vs. Chow, P< 0.01, HFD_hiSTZ vs. Chow, P < 0.05, Fig 5D).

## Discussion

The present study has utilised the C57BL/6 mouse to characterise models of diabetes and obesity, and thereafter demonstrate their renal characteristics. In particular, normal diet together with five low doses of STZ resembles T1D with marked hyperglycaemia and functional and structural renal changes. In comparison, HFD feeding induces many features of the metabolic syndrome including increased body weight, adiposity, hyperinsulinaemia, insulin resistance, hyperlipidaemia and glucose intolerance. Renal effects include functional changes demonstrated by albuminuria and higher serum creatinine. Marked structural changes distinct from the normal kidney were seen in the HFD-fed animals including glomerulosclerosis and tubular injury. The addition of a single dose of STZ together with HFD mitigates the effect of HFD on adiposity and hyperinsulinaemia. There was no lasting effect on glucose tolerance beyond that induced by HFD alone by Week 32. In addition, there was very little added benefit of one dose of STZ on renal changes, particularly structural changes induced by HFD. These results suggest that the short-term insult of one dose of STZ is not sufficient to worsen either the metabolic or the renal changes seen by HFD alone.

The model of multiple low doses of STZ employed in this study using the C57BL/6 strain adequately demonstrated the key features of T1D including a lean phenotype, hypoinsulinaemia and hyperglycaemia as demonstrated by both raised fasting glucose and HbA1c. The kidney damage seen in the Chow_lowSTZ group was sufficient to demonstrate functional changes, as evidenced by increased urinary ACR and serum creatinine. Moreover, the renal structural changes were in keeping with known histological changes associated with diabetic nephropathy in relation to tubular injury [30]. Of interest is the finding of glycogenated nuclei seen only in the Chow_lowSTZ group. Nuclear inclusion bodies are rarely seen on histological examination and are the result of the accumulation of substances not normally found in the nucleus, one of which includes glycogen [31,32]. Kang and colleagues previously described marked glycogenated nuclei in the rat kidney several months after induction of diabetes with alloxan [33]. Moreover, in a rat model of STZ-induced diabetes, hyperglycaemia was associated with large glycogen deposits in renal tubular cells nine months later [34]. The presence of nuclear inclusion bodies is likely to reflect hyperglycaemia and lead to cellular damage [35]. In our study, the histopathological changes seen in the Chow_lowSTZ group were clearly juxtaposed against those changes seen in the HFD and HFD_hiSTZ groups.

T2D is characterised by insulin resistance and β-cell failure [36]. The C57BL/6 mouse strain is highly susceptible to the metabolic effects of high fat feeding and this model has the advantage of being easily accessible [26]. The concept of using STZ to provide the “second hit” to reduce β-cell mass and further emulate T2D has been previously explored [24,37,38]. Of note, Gilbert *et al*. found that islet mass was not affected by diet but was reduced by 50% in mice that received STZ injections. Furthermore, another study demonstrated that one dose of STZ with HFD resulted in metabolic features of T2D and induced interstitial fibrosis and glomerulosclerosis in the kidney [38]. They further found evidence of renal lipotoxicity, inflammation and oxidative stress. However in this study, one dose of STZ in addition to HFD feeding did not show significant advantage to model diabetic renal pathology. In addition, the HFD_hiSTZ group was less obese than the HFD alone group. We speculate that increased glomerulosclerosis is a result of HFD induced obesity/metabolic syndrome, which was less evident in the STZ treated mice. Furthermore, the HFD_hiSTZ group did not have elevated HOMA-IR (evidence of insulin resistance). We postulate that it is due to the suppressive effect of STZ on β-cell production of insulin, thereby preventing increased insulin secretion. Thus, high insulin levels were not observed in the HOMA-IR test for this group of animals.

Obesity-propagated metabolic syndrome is associated with increased visceral adiposity, hyperlipidaemia, hypertriglyceridaemia and insulin resistance; all key features that were most clearly demonstrated in the HFD group in the present study. Blood pressure is also known to be elevated in HFD-fed animals [39]. The glucose tolerance test is an important indicator of glucose intolerance and diabetes in rodent models and the test is essential when carrying out metabolic research using rodents [40]. Our results demonstrate that glucose tolerance becomes severely impaired as the mice become older and more obese on HFD irrespective of treatment with STZ. Interestingly, HbA1c was not elevated in either group fed HFD despite significant glucose intolerance demonstrated by IPGTT. It has been suggested that 6-h fasting glucose correlates more closely with HbA1c than overnight-fasted blood glucose [27]. Indeed, the baseline glucose levels during IPGTT (after 6-h fasting) were similar among groups.

The effect of HFD in the wild type mouse on body habitus and renal damage is insidious and is due to a multitude of pathophysiologic changes including hormonal, metabolic and vascular effects driven by changes in inflammation, oxidative stress and endothelial function [41]. Though genetically manipulated models can be useful for interrogating a particular aspect of renal pathophysiology, they are less useful for exploring the whole body effects of metabolic syndrome and associated kidney disease. Although HFD feeding in C57BL/6 mice did not cause profound diabetic nephropathy in previous studies, our study shows sufficient renal damage to offer this model as a useful one when examining metabolic syndrome and renal outcomes. The length of the modelling, 24 weeks, may be the key to inducing such meaningful renal changes due to obesity and insulin resistance.

The most striking feature seen in the kidneys of the HFD group was tubular vacuolation and tubular dilatation with moderate effects on the glomerular structure. This observation of tubular vacuolisation has been reported previously in the setting of HFD-induced obese mouse models and is associated with CKD [42–45]. In fact, this finding has been shown to relate to lysosome accumulation secondary to altered lysosomal system function and altered lipid metabolism characterised by cholesterol and phospholipid accumulation in the kidney [43]. Tubular lipid accumulation has been previously shown to relate to CKD development in both humans and mice [46,47]. There was certainly evidence of hyperlipidaemia, hypertriglyceridaemia and hepatomegaly seen in the HFD group in the present study, and we have shown increase kidney lipid deposition in a rat model of obesity using the same diet [28,48]. Furthermore, abnormal autophagy in the setting of obesity has been implicated to lead to tubulointerstitial changes which may also have played a role in the tubular abnormalities seen [44].

In summary, the use of HFD in the C57BL/6 mouse is a suitable model to induce whole body and metabolic effects commonly seen in the human metabolic syndrome and is associated with renal damage likely to lead to progressive renal disease. The use of one dose STZ in addition to HFD does not provide added advantage in terms of either metabolic or renal characteristics related to T2D. The T1D model utilising five doses of STZ to induce insulin deficiency over a time interval of 24 weeks is associated with significant functional and structural renal damage. Therefore, both the T1D and HFD models using the C57BL/6 mouse strain are simple, effective models by which to understand diabetes and obesity related kidney disease and develop new diagnostic and treatment strategies.
